# Non-invasive Brain Stimulation for Gambling Disorder: A Systematic Review

**DOI:** 10.3389/fnins.2020.00729

**Published:** 2020-08-18

**Authors:** Chiara Zucchella, Elisa Mantovani, Angela Federico, Fabio Lugoboni, Stefano Tamburin

**Affiliations:** ^1^Neurology Unit, Department of Neurosciences, Verona University Hospital, Verona, Italy; ^2^Department of Neurosciences, Biomedicine and Movement Sciences, University of Verona, Verona, Italy; ^3^Addiction Medicine Unit, Department of Medicine, Verona University Hospital, Verona, Italy

**Keywords:** behavioral addiction, gambling disorder, non-invasive brain stimulation, transcranial direct current stimulation, transcranial electrical stimulation, transcranial magnetic stimulation

## Abstract

**Background:** Gambling disorder (GD) is the most common behavioral addiction and shares pathophysiological and clinical features with substance use disorders (SUDs). Effective therapeutic interventions for GD are lacking. Non-invasive brain stimulation (NIBS) may represent a promising treatment option for GD.

**Objective:** This systematic review aimed to provide a comprehensive and structured overview of studies applying NIBS techniques to GD and problem gambling.

**Methods:** A literature search using Pubmed, Web of Science, and Science Direct was conducted from databases inception to December 19, 2019, for studies assessing the effects of repetitive transcranial magnetic stimulation (rTMS) and transcranial direct current stimulation (t-DCS) on subjects with GD or problem gambling. Studies using NIBS techniques on healthy subjects and those without therapeutic goals but only aiming to assess basic neurophysiology measures were excluded.

**Results:** A total of 269 articles were title and abstract screened, 13 full texts were assessed, and 11 were included, of which six were controlled and five were uncontrolled. Most studies showed a reduction of gambling behavior, craving for gambling, and gambling-related symptoms. NIBS effects on psychiatric symptoms were less consistent. A decrease of the behavioral activation related to gambling was also reported. Some studies reported modulation of behavioral measures (i.e., impulsivity, cognitive and attentional control, decision making, cognitive flexibility). Studies were not consistent in terms of NIBS protocol, site of stimulation, clinical and surrogate outcome measures, and duration of treatment and follow-up. Sample size was small in most studies.

**Conclusions:** The clinical and methodological heterogeneity of the included studies prevented us from drawing any firm conclusion on the efficacy of NIBS interventions for GD. Further methodologically sound, robust, and well-powered studies are needed.

## Introduction

Gambling disorder (GD), also known as pathological gambling, affects people of all ages and is a major clinical issue associated with reduced quality of life, psychiatric comorbidity, cognitive deficits, and higher risk of suicide (Ledgerwood and Petry, 2004; Hodgins et al., 2011; Nautiyal et al., 2017). GD and other impulse control disorders (ICDs) are also common in patients with Parkinson's disease (PD) under dopaminergic treatment (Antonini et al., 2017).

Gambling disorder (GD) was previously classified as an ICD but is currently considered the prototypical example of behavioral addiction and is included in the diagnostic category of substance-related and addictive disorders according to the *Diagnostic and Statistical Manual of Mental Disorders*, fifth edition (*DSM-5*; American Psychiatric Association, 2013; Nautiyal et al., 2017), because a growing evidence suggests that GD and substance use disorder (SUD) share common neurobiological bases and behavioral features (Hudgens-Haney et al., 2013; Limbrick-Oldfield et al., 2013; Tschernegg et al., 2013; Goudriaan et al., 2014). Indeed, human and animal studies indicate that both GD and SUD are characterized by a dysfunction of the reward and cognitive control systems, leading to craving, altered sensitivity to reward, reduced self-control, and abnormal decision-making and executive function (Koob and Volkow, 2016).

The impairment of dopaminergic brain reward circuitries, which are supposed to play a key role in SUD (Vaughan and Foster, 2013), has been reported in GD (Clark et al., 2019). Reduced striatal dopamine transporter availability (Pettorruso et al., 2019b) and increased dopamine synthesis capacity (van Holst et al., 2018) were reported in GD compared with healthy controls. Similarly, PD patients with GD and ICD after dopaminergic treatment showed lower dopaminergic transporter levels in the dorsal striatum and increased dopamine release in the ventral striatum when engaged in reward-related stimuli/gambling tasks (Martini et al., 2018a). An image-based meta-analysis documented striatal hypoactivation in patients with SUD during reward anticipation and in those with GD during reward outcome, in line with the reward-deficiency theory of addiction (Luijten et al., 2017). According to the learning-deficit model (Luijten et al., 2017), these abnormalities are supposed to sustain the transition toward compulsive gambling addiction, characterized both by hypodopaminergic and hyperdopaminergic states in the context of a sensitized dopaminergic system (Pettorruso et al., 2019b).

Executive dysfunction documented in GD patients suggests the involvement of the cognitive control system that can be differentiated into several cognitive sub-processes, i.e., response inhibition, conflict monitoring, decision making, and cognitive flexibility (Moccia et al., 2017). Human functional neuroimaging studies have shown changes in prefrontal regions leading to diminished cognitive control pivotal to the development of GD (Moccia et al., 2017). The cognitive control circuit includes the median prefrontal cortex (mPFC), the dorsolateral prefrontal cortex (DLPFC), the orbital and ventromedial areas, and the anterior cingulate cortex (Van Holst et al., 2010). PD patients with GD and ICD show worse set-shifting and reward-related decision-making and increased depression, anxiety, anhedonia, and impulsivity, pointing to more severe executive dysfunction (Martini et al., 2018b).

GD is considered a full-fledged worldwide public health concern because of its detrimental individual, social, and economic consequences and reduced quality of life (Williams et al., 2012). Moreover, the number of “at risk gamblers” (i.e., people who gamble frequently but not yet pathologically dependent) is increasing (Cavalera et al., 2018). A comprehensive systematic review of empirical researches from 2000 to 2015 across different countries in the world showed that 0.1–5.8% of individuals met diagnostic criteria for problem gambling during the year before the survey and 0.7–6.5% for problem gambling during their lifetime (Calado and Griffiths, 2016). In addition, a recent study estimated the prevalence of GD in Italy to range from 1.3 to 2.2%, and that of “at risk gamblers” to be 1.3–3.8% (ISS, 2018).

Because of the absence of pharmacological treatments with proven efficacy for this condition, the role of non-invasive brain stimulation (NIBS) has been explored for the treatment of GD and other behavioral addictions (Sauvaget et al., 2015). Repetitive transcranial magnetic stimulation (rTMS) and transcranial direct current stimulation (t-DCS) are the most commonly used types of NIBS.

rTMS is delivered to the brain by a rapid phasic electrical current through an insulated wire coil placed over the skull that generates a transient magnetic field, which propagates in space and induces secondary currents that may depolarize neurons in targeted brain regions and lead to neuromodulation and neuroplastic changes (Pascual-Leone et al., 1998; Paulus et al., 2013). High-frequency rTMS is excitatory, while low-frequency rTMS decreases cortical excitability (Paulus et al., 2013), but the effects on synaptic plasticity are often weak, highly variable between individuals, and short-lasting (Huang et al., 2005). Theta-burst stimulation (TBS) is a modified rTMS type that has been found to produce a consistent, long-lasting, and powerful effect on cortex physiology and behavior, with a mixture of facilitatory and inhibitory effects on synaptic transmission according to the TBS protocol used (i.e., prevalent facilitation to intermittent TBS and prevalent inhibition to continuous TBS; Huang et al., 2005). Spatial and temporal resolution of rTMS are high and the former may be modified by the type of coil, with the classical figure-of-eight coil providing superficial and focal stimulation, and more recent H-coils able to target brain regions to a depth of 5–7 cm (Rossi et al., 2009). The main side effects of rTMS are transient scalp discomfort, headache, and hearing disorders, usually following high frequency protocols (Rossi et al., 2009). The risk of inducing epileptic seizures is minimized through the application of the guidelines and an accurate selection of patients (Lefaucheur et al., 2014, 2020).

t-DCS is delivered through a battery-powered device connected to a couple of electrodes that deliver low-amplitude direct intracerebral currents that increase or decrease neuronal excitability in the specific brain area being stimulated through modification of membrane polarization (Nitsche and Paulus, 2000). Generally, anodal t-DCS depolarizes neurons, thus increasing cortical excitability, whereas cathodal t-DCS hyperpolarizes neurons, reducing cortical excitability (Nitsche and Paulus, 2000). When applied for a sufficient period of time, t-DCS induces sustained changes in cortical excitability (Nitsche and Paulus, 2001; Nitsche et al., 2005). t-DCS is usually safe and may cause only mild side effects, such as burning sensation and skin irritation, especially with daily use or higher current intensity (Antal et al., 2017), but its spatial and temporal resolution is limited.

Recommendations and guidelines for the safe and appropriate application of NIBS for clinical and research application have been published (Rossi et al., 2009; Rossini et al., 2015; Woods et al., 2016). Guidelines on the therapeutic use of NIBS proposed level A recommendation (definite efficacy) for rTMS of the left dorsolateral prefrontal cortex (DLPFC) in the treatment of depression (Lefaucheur et al., 2020) and level B recommendation (probable efficacy) for anodal tDCS of the left DLPFC (with right orbitofrontal cathode) in major depressive episode without drug resistance and anodal tDCS of the right DLPFC (with left DLPFC cathode) in addiction/craving (Lefaucheur et al., 2017). Recent studies highlighted the potential of rTMS for some SUDs (Diana et al., 2017).

Data on the therapeutic options for GD are scarce. Moreover, information on potentially effective treatments for this condition are needed, because of its social and economic impact. Since the application of NIBS to GD is a very recent field of interest, the present manuscript is aimed to offer a systematic review on studies applying rTMS and t-DCS to patients with GD.

## Methods

This systematic review was conducted according to the Preferred Reporting Items for Systematic Reviews and Meta-Analyses (PRISMA) recommendations (Liberati et al., 2009; Moher et al., 2015).

### resEligibility Criteria

We included studies assessing the effects of NIBS techniques in subjects with a diagnosis of GD or pathological/problem gambling. Both controlled and exploratory studies were included and considered eligible, and no restrictions were placed on the publication date of the studies.

We excluded reviews, commentaries, letters, abstracts, conference papers, and animal model studies. Studies applying NIBS techniques on healthy subjects were also excluded. Studies considering NIBS techniques without therapeutic goals but only aiming at assessing basic neurophysiology measures were not considered eligible.

Primary outcomes of interest were changes in clinical (i.e., GD severity, craving, relapse, abstinence, psychiatric related symptoms) or para-clinical outcomes (i.e., physiological measures).

### Search Strategy

The Pubmed, Science Direct, and Web of Science databases were searched for peer-reviewed studies on NIBS techniques in subjects with/or at risk of GD or pathological/problem gambling and published from databases inception until December 19, 2019. Only studies written in English were considered.

The search string for Pubmed and Web of Science was: (gambling disorder OR pathological gambling OR problem gambling OR compulsive gambling OR gambling addiction OR gambling addictions OR problematic gambling OR pathological gamblers OR problem gamblers OR gamblers anonymous OR gambling addicts OR gambling) AND (transcranial magnetic stimulation OR TMS OR r-TMS OR theta burst stimulation OR theta burst OR TBS OR c-TBS OR i-TBS OR NIBS OR non-invasive brain stimulation OR brain stimulation OR transcranial direct current stimulation OR tDCS OR tES OR transcranial electrical stimulation OR tCS OR transcranial current stimulation).

The search strategy for Science Direct database included: (Gambling OR gamblers) AND (NIBS OR non-invasive brain stimulation OR brain stimulation), then (Gambling OR gamblers) AND (transcranial magnetic stimulation OR TMS OR r-TMS OR theta burst stimulation OR TBS OR c-TBS OR i-TBS), and (Gambling OR gamblers) AND (transcranial direct current stimulation OR tDCS OR tES OR transcranial electrical stimulation OR tCS OR transcranial current stimulation).

### Study Selection

Two authors (CZ and EM) independently screened titles and abstracts using Rayyan software (Ouzzani et al., 2016). The reference lists of relevant papers were inspected for additional studies potentially missed in the databases search. Any disagreement was planned to be solved by consensus or consulting a third reviewer (ST).

### Data Collection Procedure

Two reviewers (CZ and EM) independently extracted the following data: study design (i.e., randomized, crossover, parallel, open label, single arm trials), sample size, gender, presence of any comorbidity with GD (i.e., psychiatric conditions, SUD), type of rTMS/t-DCS protocol (excitatory/inhibitory effect, session numbers, blinding, sham condition, side effects, follow-up duration), targeted brain area, outcomes of interest (i.e., clinical, surrogate).

### Data Analysis

A descriptive analysis of the results was carried out, focusing on the effects of the interventions. A meta-analysis was not possible due to the small number of studies and subjects, and to the clinical, methodological (NIBS protocol, brain target), and outcome heterogeneity of the included studies.

## Results

### Identification and Selection of the Studies

A total of 400 records were identified. After removal of duplicates, 269 papers were screened through title and abstract and 13 papers were obtained for full-text screening. The reference lists of the relevant papers were inspected for additional studies potentially missed in the databases search, but no significant papers were further added. Two authors (CZ and EM) independently evaluated the 13 papers selected for the full-text examination. Disagreement was solved by consensus between the two reviewers, therefore the third reviewer's (ST) advice was not required.

**Figure 1:**
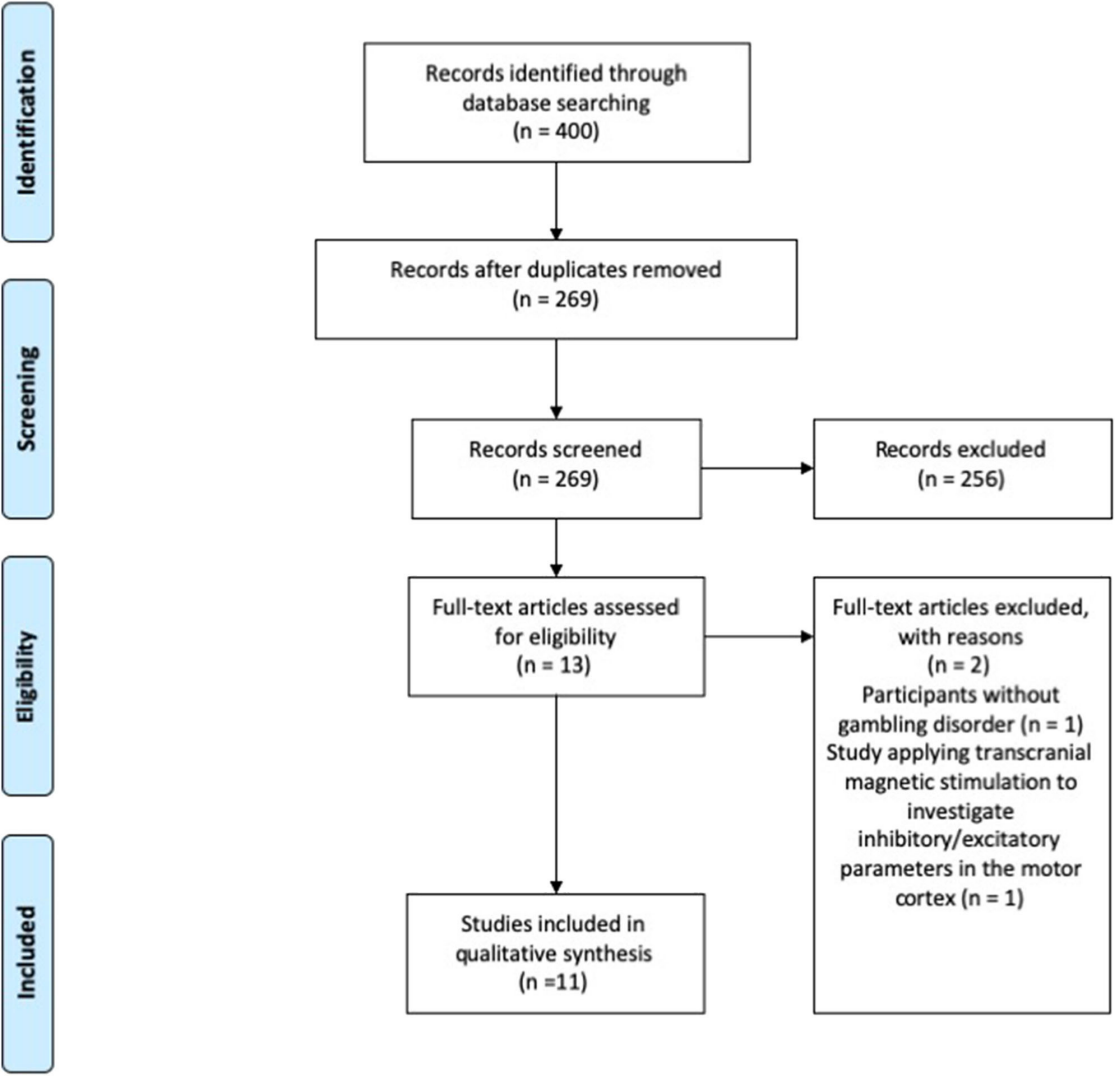
PRISMA diagram of the study (www.prisma-statement.org).

Eleven studies met the inclusion criteria and were therefore included in the systematic review (Figure 1).

### Description of the Included Studies

The included papers evaluated the efficacy of NIBS interventions based on rTMS or t-DCS techniques for subjects with GD or problem gambling. Studies were grouped according to the NIBS technique employed (i.e., rTMS or t-DCS) and the presence or absence of a sham-NIBS control arm.

### rTMS Studies

Seven studies employed rTMS in GD (Table 1).

**Table 1:** Overview of rTMS studies included in the review.

**References**	**Study design**	**Population**	**Sample size**	**Stimulation site**	**Stimulation protocol**	**Outcome measures (clinical)**	**Outcome measures (surrogate)**	**Follow-up**	**Side effects**	**Results**
Rosenberg et al. (2013)	Open label	GD patients (men, age 37.8 ± 10.5)	5	Left DLPFC	15 days, one session/day, 10 min duration, 1 Hz, 110% motor threshold	HDRS, HARS, Y-BOCS, SOGS, DAGS, VAS, CGI–I, SAS^1^	None	Interview to families	None	HDRS, HARS, CGI, Y-BOCS, and VAS improved after treatment; all patients returned to gamble
Zack et al. (2016)	Sham-controlled cross-over	Community-recruited, non-treatment seeking men with GD (age 43.2 ± 13.2)	9	mPFC, right DLPFC	rTMS, 10 Hz, 450 pulses (mPFC) cTBS, 50 Hz, 900 pulses (DLPFC) Three sessions (rTMS, cTBS, sham; 1-week washout)	Desire to gamble (VAS)	DDT, Stroop task, arousal (blood pressure, POMS-vigor scale, ARCI)	None	None	rTMS reduced desire to gamble; cTBS reduced amphetamine-like effects and diastolic blood pressure
Gay et al. (2017)	Randomized sham-controlled cross-over	GD patients	22	Left DLPFC	High frequency rTMS (single session)	Gambling craving, PG-YBOCS	None	None	None	Decrease in gambling craving
Sauvaget et al. (2018)	Randomized double-blind sham-controlled cross-over	Men with GD seeking treatment (age range 28–56)	30	Right DLPFC	rTMS, 1 Hz, 360 pulses Two sessions (active, sham; 1-week washout)	Craving (VAS, GCS)	Heart rate, blood pressure	None	None	Both active and sham rTMS were associated with significant decrease in the urge to gamble
Cardullo et al. (2019)	Case series	Men with GD and cocaine use disorder (mean age 42.1 ± 5.7)	7	Left DLPFC	Twice/day for 5 consecutive days, then twice/day once a week for 8 weeks, 15 Hz, 100% motor threshold, 60 pulses per train, 40 trains, 15 s inter-train interval, 13 min duration	G-SAS, CCQ, PSQI, BDI—II, SAS^2^, GSI	None	At day 5, 30, at 60 after the beginning of treatment	None	Improvement of gambling severity, cocaine craving, and negative-affect symptoms; results stable at follow-ups
Pettorruso et al. (2019b)	Case report	One man with GD (40 years)	1	Left DLPFC	Twice/day for 5 days/week for 2 weeks (20 sessions), then twice/daily, one/week for 12 weeks (24 sessions), 15 Hz, 100% motor threshold, 60 pulses per train, 40 trains, 15 s inter-train interval, 2,400 pulses/session, 13 min duration	BDI, G-SAS, PG-YBOCS, ISI, YMRS	DAT availability measured by SPECT	One and two weeks, one, two, three, six months	None	No craving for gambling or gambling-related symptoms; decrease in DAT availability in striatal regions
Pettorruso et al. (2020)	Open-label feasibility study	GD patients (age 40.6 ± 11.2)	8 (7 men)	Left DLPFC	Twice/day for 5 days/week for 2 weeks (20 sessions), then twice/daily, one/week for 12 weeks (24 sessions), 15 Hz, 100% motor threshold, 60 pulses per train, 40 trains, 15 s inter-train interval, 2,400 pulses/session, 13 min duration	G-SAS, PG-YBOCS, GTFB, BDI, SAS^2^	None	Two, four, eight, and 12 weeks	None	Reduction of gambling behavior and the number of days spent gambling; results confirmed during all the follow-up period

*ARCI, Addiction Research Center Inventory; BDI, Beck depression inventory; BDI-II, Beck depression inventory-II; CCQ, Cocaine craving questionnaire; CGI-I, Clinical global impression improvement scale; cTBS, continuous theta burst stimulation; DAGS, Dannon and ainhold gambling scale; DAT, Dopamine active transporter; DDT, Delay discounting task; DLPFC, Dorsolateral prefrontal cortex; GCS, Gambling craving scale; GD, Gambling disorder; G-SAS, Gambling symptom assessment scale; GSI, Global severity index; GTSB, Gambling timeline follow back; HARS, Hamilton anxiety rating scale; HDRS, Hamilton depression rating scale; ISI, Insomnia severity index; mPFC, medial prefrontal cortex; PG-YBOCS, Pathological gambling adaptation of the Yale-Brown obsessive-compulsive scale; POMS, Profile of mood states; PSQI, 19-item Pittsburgh sleep quality index; rTMS, repetitive transcranial magnetic stimulation; SAS^1^, Social adjustment scale; SAS^2^, Self-rating anxiety scale; SOGS, South oaks gambling screen; SPECT, Single photon emission computed tomography; VAS, Visual analog scale; Y-BOCS, Yale-Brown obsessive compulsive scale; YMRS, Young mania rating scale*.

#### Controlled Studies

Zack et al. (2016) assessed the effect of two rTMS protocols on gambling reinforcement and related responses in nine community-recruited, non-treatment-seeking men with GD. They reported that three sessions of high frequency rTMS targeting mPFC yielded a significant reduction of craving, in particular in the post-game increase in the desire to gamble, and that the same sessions number of continuous TBS targeting the right DLPFC reduced amphetamine-like effects (i.e., psychostimulant-like sensations measured with the Addiction Research Center Inventory amphetamine scale) and behavioral activation measured with diastolic blood pressure, but no changes were reported in impulsive choices or cognitive control on the Stroop task (Zack et al., 2016).

Gay et al. (2017) performed a randomized sham-controlled cross-over study on 22 GD patients using a single session of high frequency rTMS over the left DLPFC and documented a decrease in cue-induced craving and no effect on gambling behavior to real rTMS, but the absence of follow-up impeded to measure the duration of the effect.

In the study conducted by Sauvaget et al. (2018), one session of low frequency rTMS targeting the right DLPFC did not lead to a significant reduction of craving, measured with both self-report scales and physiological measures, compared to sham stimulation.

#### Uncontrolled Studies

In an open-label study that explored the effect of 15 sessions of low frequency rTMS over the left DLPFC in five participants with GD, despite initial improvement in rating scales, the effect decayed over time and the authors concluded that rTMS treatment failed to demonstrate effectiveness (Rosenberg et al., 2013).

Cardullo et al. (2019) evaluated the effect of 26 sessions of high-frequency rTMS over the left DLPFC in seven men with dual diagnosis of GD and cocaine use disorder and found significant improvement in gambling severity, cocaine craving, and negative-affect symptoms compared to baseline.

Pettorruso et al. (2019a) described a GD patient who was treated with 44 sessions of high frequency rTMS over the left DLPFC and reported a marked reduction in craving for gambling and no episodes of gambling during the 6-month follow-up. Of note, the authors found decreased dopamine transporter availability, a neurobiological marker of dopaminergic pathways modulation, after 2 weeks of treatment.

The same authors investigated eight GD treatment-seeking patients treated with 44 sessions of high frequency rTMS targeting the left DLPFC in an open-label study that showed significant reduction of gambling episodes and the days of gambling throughout the study period in comparison to baseline (Pettorruso et al., 2020).

### t-DCS Studies

Four studies employed t-DCS in GD (Table 2).

**Table 2:** Overview of t-DCS studies included in the review.

**References**	**Study design**	**Population**	**Sample size**	**Stimulation site**	**Stimulation protocol**	**Outcome measures (clinical)**	**Outcome measures (surrogate)**	**Follow-up**	**Side effects**	**Results**
Martinotti et al. (2018)	Case report	One man with GD and alcohol and cocaine use disorder, 26 years	1	Left and right DLPFC	Twice/day, 20 min stimulation, 1.5 mA, 1-h interval between left and right DLFPFC, 10 consecutive days	SOGS, BPRS, HDRS, HARS, BIS, VAS, PG-YBOCS, G-SAS	None	Ten, 100, and 190 days after t-DCS	None	Significant improvement of gambling, craving severity, and psychiatric symptoms; further improvement at follow-ups
Dickler et al. (2018)	Randomized double-blind sham-controlled crossover	Patients with GD (age range 21–65)	16 (9 men, 7 women)	DLPFC	Anode on right DLPFC, cathode on left DLPFC, two sessions, 1 mA, 30 min (active, sham; 1-week washout)	Craving (GCS)	Metabolite levels (GABA, Glx, NAA), BART, BIS	None	None	Active t-DCS increased GABA levels compared to sham, positive correlations with BART, BIS, GCS
Soyata et al. (2019)	Randomized triple-blind sham-controlled parallel design	Patients with GD (age range 18–55)	20	DLPFC	Anode on right DLPFC, cathode on left DLPFC, three session, 2 mA, 20 min (active, sham)	Not assessed	IGT, WCST, Stroop task	None	None	Active t-DCS yielded better performance at WCST and Stroop task compared to sham
Martinotti et al. (2019)	Randomized, double-blind sham-controlled parallel design	Treatment-seeking GD subjects	34	DLPFC	Anode on right DLPFC, cathode on left DLPFC, five consecutive sessions (active, sham)	Craving (VAS)	None	None	None	Active t-DCS significantly reduced craving levels compared to sham

*BIS, Barratt impulsiveness scale; BART, Balloon analog risk taking task; BPRS, Brief psychiatric rating scale; DLPFC, Dorsolateral prefrontal cortex; GABA, Gamma-aminobutyric acid; GCS, Gambling craving scale; GD, Gambling disorder; Glx, Glutamine-glutamate-GABA complex; G-SAS, Gambling symptom assessment scale; HARS, Hamilton anxiety rating scale; HDRS, Hamilton depression rating scale; IGT, Iowa gambling task; NAA, N-acetyl aspartate; PG-YBOCS, Pathological gambling adaptation of the Yale-Brown obsessive-compulsive scale; SOGS, South oaks gambling screen; t-DCS, Transcranial direct current stimulation; VAS, Visual analog scale; WCST, Wisconsin card sorting test; Y-BOCS, Yale-Brown obsessive compulsive scale*.

#### Controlled Studies

Dickler et al. (2018) used a montage to administer anodal t-DCS on the right DLPFC and cathodal t-DCS on the left DLPFC to characterize its effects on neural metabolites levels measured with magnetic resonance spectroscopy. They found that two sessions of active t-DCS induced significantly increased GABA levels in comparison to sham t-DCS, and that metabolite levels were positively correlated with measures of risk taking, impulsivity, and craving (Dickler et al., 2018).

Soyata et al. (2019) reported that three sessions of active anodal t-DCS on the right DLPFC and cathodal t-DCS on the left DLPFC modulated decision making and cognitive flexibility, leading to more advantageous choices during the Iowa Gambling Task and better performances at the Wisconsin Card Sorting Test in participants with GD.

Martinotti et al. (2019) reported that five consecutive sessions of active anodal right DLPFC t-DCS induced a significant reduction of craving levels in comparison to sham t-DCS in a group of treatment-seeking GD patients.

#### Uncontrolled Studies

Martinotti et al. (2018) reported a young male with 8-year history of GD comorbid with alcohol and cocaine use disorder who was treated with 20 sessions of bilateral DLPFC t-DCS and showed improvement of psychiatric symptoms (depression, anxiety, and impulsivity) and gambling craving, which were maintained at follow-up visits.

## Discussion

This systematic review explored the effect of rTMS and t-DCS in people affected by GD. We have found a small number of studies, i.e., seven rTMS and four t-DCS studies, and among them only six were controlled ones, i.e., three on rTMS and three on t-DCS, while the other five reports had an uncontrolled design or were case reports/series.

Despite some differences among outcome measures, most controlled studies (Zack et al., 2016; Gay et al., 2017; Martinotti et al., 2019) and uncontrolled reports (Martinotti et al., 2018; Cardullo et al., 2019; Pettorruso et al., 2019a, 2020) reported a reduction of gambling behavior, craving, or gambling-related symptoms, while the effect on coexistent psychiatric symptoms (e.g., depression, anxiety) was less consistent. Notably, one controlled (Sauvaget et al., 2018) and one uncontrolled study (Rosenberg et al., 2013) reported no changes to rTMS. Two controlled t-DCS studies found improvement in surrogate outcome measures, namely brain gamma-aminobutyric acid levels (Dickler et al., 2018) and neuropsychological testing scores (Soyata et al., 2019), but no improvement in clinical measures.

Taken together, the current evidence lends very limited support to the use of NIBS in patients with GD. It should be noted that the papers we have included in the systematic review were quite heterogeneous in terms of study design, study population, outcome measures, duration of follow-up, comorbidities, all factors that hampered a meta-analytical approach. Moreover, only three studies were comparable in terms of stimulation protocol features and brain target (Dickler et al., 2018; Martinotti et al., 2019; Soyata et al., 2019), but the outcome measures were heterogeneous and impeded a meta-analysis.

All studies targeted the DLPFC, but they were not consistent in terms of brain side, and one study targeted also the mPFC (Zack et al., 2016). The rationale of choosing the DLPFC is because this target is a key structure in the cognitive control circuit (Moccia et al., 2017), which is supposed to be altered in GD patients, leading to compulsive gambling, craving, impaired reward sensitivity, self-control, and decision-making processes (Van Holst et al., 2010; Koob and Volkow, 2016). Moreover, changes in impulsivity and risky decision-making have been reported after the application of rTMS or t-DCS over prefrontal regions in healthy subjects (Fecteau et al., 2007a,b; Cho et al., 2010; Lantrip et al., 2017). Among the studies we included, however, only two t-DCS reports explored behavioral or neuropsychological measures (Dickler et al., 2018; Soyata et al., 2019).

The studies differed for the site of stimulation, with five rTMS reports targeting the left DLPFC, and two targeting the right one. Conversely, all t-DCS studies targeted both the left and the right DLPFC. The rationale for the left DLPFC preference in rTMS studies may result from studies on SUD, where rTMS over the left DLPFC was reported to be effective in reducing craving, enhancing cognitive control (Politi et al., 2008; Jansen et al., 2013; Rapinesi et al., 2016; Terraneo et al., 2016), and in improving cognitive functioning (Schluter et al., 2018) and the supposed pathophysiological communalities between GD and SUD (Hudgens-Haney et al., 2013; Limbrick-Oldfield et al., 2013; Tschernegg et al., 2013; Goudriaan et al., 2014). Two studies applied rTMS to the right DLPFC and found no improvement in clinical outcomes but some changes in autonomic measures (Zack et al., 2016; Sauvaget et al., 2018). Despite being very preliminary and based on a small number of patients, these data may suggest a preference for the left DLPFC. However, rTMS of the prefrontal regions has been demonstrated to induce bilateral changes in the pattern of brain activation, because of the activation of monosynaptic afferents in the contralateral hemisphere or the influence on functional connectivity patterns of bilateral frontostriatal circuits (Hanlon et al., 2013; Schluter et al., 2017). Because of these concerns, the laterality issue for rTMS of the DLPFC should be further explored in future studies.

In t-DCS studies, DLPFC was targeted bilaterally, either separately in two sessions the same day (Martinotti et al., 2018) or together in the same session through the application of the anode over the right DLPFC and the cathode over the left one (Dickler et al., 2018; Martinotti et al., 2019; Soyata et al., 2019). The choice of this stimulation protocol was based on previous reports that these parameters were associated with a reduction of spontaneous (Batista et al., 2015; Klauss et al., 2018) and cue-induced craving (Fregni et al., 2008a,b; Boggio et al., 2010) and impulsivity (Fecteau et al., 2007a,b; He et al., 2016; Shen et al., 2016; Soff et al., 2017) in patients with SUD and attention deficit hyperactivity disorder.

Stimulation parameters were also not consistent across studies. Two rTMS studies, which used low frequency rTMS (i.e., inhibitory effect), reported no significant changes (Rosenberg et al., 2013; Sauvaget et al., 2018). Conversely, three studies used high frequency rTMS (i.e., excitatory effect) and found significant results (Gay et al., 2017; Cardullo et al., 2019; Pettorruso et al., 2019a, 2020). A single rTMS study compared excitatory high frequency rTMS over the mPFC to inhibitory continuous TBS over the right DLPFC and found differential effects among the two types of NIBS (Zack et al., 2016). Taken together these findings would favor high frequency rTMS for future studies. Studies varied also in terms of the duration of rTMS from a single session to multiple days up to 8 weeks. The very short follow-up periods, which were often limited to the time of rTMS application, impede us from drawing any conclusion whether the changes may outlast the treatment period.

Three out of the four reports on t-DCS used excitatory anodal t-DCS over the right DLPFC and inhibitory cathodal t-DCS over the left one, impeding any conclusion on whether the effects in GD patients were due to excitation or inhibition of the DLPFC.

All studies reported no side effects, confirming the overall safety of NIBS techniques when studies are conducted according to the safety and application guidelines (Rossi et al., 2009; Rossini et al., 2015; Woods et al., 2016; Antal et al., 2017).

Several limitations may have contributed to the inconsistencies across the studies we reviewed. First, all studies had small sample sizes ranging from single case reports to 30–34 patients, with a large majority of men, hampering the generalization of the findings to larger and gender-balanced populations of patients (Ekhtiari et al., 2019; Luigjes et al., 2019). Second, the heterogeneity of the type of stimulation (i.e., excitatory, inhibitory) and duration of stimulation sessions impede any conclusions on the optimal stimulation parameters. Third, targeted brain areas and site varied across studies, with most of them focusing on the DLPFC, despite the inconsistencies on the stimulated side, because of its fundamental role on the cognitive control circuit. This target was probably chosen because of the data from SUD patients (Ekhtiari et al., 2019), and the similarities between SUD, behavioral addiction, and GD. Indeed, these conditions share common behavioral (e.g., impulsivity), neurophysiological, and brain structural and functional changes involving bilateral insula, amygdala, hippocampi, parahippocampal gyri, prefrontal cortex, and anterior cingulate cortex, but they also show some differences, especially in striatal connectivity (Gomis-Vicent et al., 2019). Moreover, studies on the neurobiology of addictive disorders indicate that the reward-related circuitry is much broader, including several other areas, such as the mPFC (Steele and Lawrie, 2004), which was targeted only in one study (Zack et al., 2016) and other subcortical areas that can be reached only with H-shaped coils (Rossi et al., 2009) that was not used in the reports we reviewed. Future studies on GD and behavioral addictions should consider the similarities and the differences between gambling and SUDs, exploring the role of NIBS on other brain areas, including the deeper ones (Spagnolo and Goldman, 2017; Gomis-Vicent et al., 2019). Fourth, another critical issue is the standardization of a panel of GD clinical outcomes together with surrogate measures that represent biomarker of changes related to NIBS. Fifth, most of the studies focused on short-term outcomes (i.e., immediate craving reduction), without adequate follow-up sessions to evaluate the persistence of changes induced by NIBS over time. Sixth, the study design may have influenced the findings. Five of the 11 studies we included were open-label ones, or case reports/series, and their conclusions should be taken with caution because of the risk of placebo effect and overstatement of the findings. Two studies used a parallel design that might have led to an increased probability of the occurrence of unblinding (Ekhtiari et al., 2019). Only three of them used a cross-over design (Zack et al., 2016; Dickler et al., 2018; Sauvaget et al., 2018) that may not be free from carry-over effects (Fregni et al., 2007; Hallett, 2007). Consensus among experts is needed to define the most appropriate study design for future studies on NIBS in GD.

## Conclusions and Future Directions

Despite the limited amount of information on the role of NIBS that prevented us to draw any conclusion on its efficacy for the treatment of GD and problem gambling, our systematic review highlighted preliminary encouraging results and provided important directions for future studies. The finding that only few studies were available on this topic, to date, in our opinion represents an interesting starting point for future research.

The studies we reviewed suggest the potential of high frequency rTMS over the DLPFC, and excitatory anodal t-DCS over the right DLPFC together with inhibitory cathodal t-DCS over the left one for GD, but these pieces of evidence should be considered still preliminary. Further larger studies should confirm these findings and address the laterality issue (i.e., targeting the left, right DLPFC, or both of them).

Another question that should be explored is whether NIBS is effective as stand-alone or add-on treatment (e.g., associated with pharmacological treatment or cognitive behavior therapy). Finally, methodologically sound and well-powered double- or triple-blind randomized controlled studies, including clinical outcomes and surrogate biomarkers, are needed to document the potential therapeutic role of NIBS in GD.

## Data Availability Statement

The datasets generated for this study are available on request to the corresponding author.

## Author Contributions

This study has been designed by CZ, EM, AF, FL, and ST. Data have been gathered by CZ and EM, under the supervision of ST. Data have been analyzed by CZ and EM. The manuscript has been drafted by CZ, EM, AF, and ST. FL and ST revised the manuscript. All authors approved the final version of the manuscript.

## Conflict of Interest

The authors declare that the research was conducted in the absence of any commercial or financial relationships that could be construed as a potential conflict of interest.

## References

[B1] American Psychiatric Association (2013). Diagnostic and Statistical Manual of Mental Disorders DSM-5, 5th Edn. Washington, DC: American Psychiatric Association 10.1176/appi.books.9780890425596

[B2] Antal A. Alekseichuk I. Bikson M. Brockmöller J. Brunoni A. R. Chen R.. (2017). Low intensity transcranial electric stimulation: safety, ethical, legal regulatory and application guidelines. Clin. Neurophysiol. 128, 1774–1809. 10.1016/j.clinph.2017.06.001 28709880 PMC5985830

[B3] Antonini A. Barone P. Bonuccelli U. Annoni K. Asgharnejad M. Stanzione P. (2017). ICARUS study: prevalence and clinical features of impulse control disorders in Parkinson's disease. J. Neurol. Neurosurg. Psychiatry 88, 317–324. 10.1136/jnnp-2016-315277 28315845

[B4] Batista E. K. Klauss J. Fregni F. Nitsche M. A. Nakamura-Palacios E. M. (2015). A randomized placebo-controlled trial of targeted prefrontal cortex modulation with bilateral tDCS in patients with crack-cocaine dependence. Int. J. Neuropsychopharmacol. 18:pyv066. 10.1093/ijnp/pyv066 26065432 PMC4675977

[B5] Boggio P. S. Zaghi S. Villani A. B. Fecteau S. Pascual-Leone A. Fregni F. (2010). Modulation of risk-taking in marijuana users by transcranial direct current stimulation of the dorsolateral prefrontal cortex. Drug Alcohol Depend. 112, 220–225. 10.1016/j.drugalcdep.2010.06.019 20729009

[B6] Calado F. Griffiths M. D. (2016). Problem gambling worldwide: an update and systematic review of empirical research (2000–2015). J. Behav. Addict. 5, 592–613. 10.1556/2006.5.2016.073 27784180 PMC5370365

[B7] Cardullo S. Gomez Perez L. J. Marconi L. Terraneo A. Gallimberti L. Bonci A.. (2019). Clinical improvements in comorbid gambling/cocaine use disorder (GD/CUD) patients undergoing repetitive transcranial magnetic stimulation (rTMS). J. Clin. Med. 8:E768. 10.3390/jcm8060768 31151221 PMC6616893

[B8] Cavalera C. Bastiani L. Gusmeroli P. Fiocchi A. Pagnini F. Molinari E.. (2018). Italian adult gambling behavior: at risk and problem gambler profiles. J. Gambl. Stud. 34, 647–657. 10.1007/s10899-017-9729-8 29134497

[B9] Cho S. S. Ko J. H. Pellecchia G. Van Eimeren T. Cilia R. Strafella A. P. (2010). Continuous theta burst stimulation of right dorsolateral prefrontal cortex induces changes in impulsivity level. Brain Stimul. 3, 170–176. 10.1016/j.brs.2009.10.002 20633446 PMC3707839

[B10] Clark L. Boileau I. Zack M. (2019). Neuroimaging of reward mechanisms in gambling disorder: an integrative review. Mol. Psychiatry 24, 674–693. 10.1038/s41380-018-0230-2 30214041

[B11] Diana M. Raij T. Melis M. Nummenmaa A. Leggio L. Bonci A. (2017). Rehabilitating the addicted brain with transcranial magnetic stimulation. Nat. Rev. Neurosci. 18, 685–693. 10.1038/nrn.2017.113 28951609

[B12] Dickler M. Lenglos C. Renauld E. Ferland F. Edden R. A. Leblond J.. (2018). Online effects of transcranial direct current stimulation on prefrontal metabolites in gambling disorder. Neuropharmacology 131, 51–57. 10.1016/j.neuropharm.2017.12.002 29221791

[B13] Ekhtiari H. Tavakoli H. Addolorato G. Baeken C. Bonci A. Campanella S.. (2019). Transcranial electrical and magnetic stimulation (tES and TMS) for addiction medicine: a consensus paper on the present state of the science and the road ahead. Neurosci. Biobehav. Rev. 104, 118–140. 10.1016/j.neubiorev.2019.06.007 31271802 PMC7293143

[B14] Fecteau S. Knoch D. Fregni F. Sultani N. Boggio P. Pascual-Leone A. (2007a). Diminishing risk-taking behavior by modulating activity in the prefrontal cortex: a direct current stimulation study. J. Neurosci. 27, 12500–12505. 10.1523/JNEUROSCI.3283-07.2007 18003828 PMC6673338

[B15] Fecteau S. Pascual-Leone A. Zald D. H. Liguori P. Theoret H. Boggio P. S.. (2007b). Activation of prefrontal cortex by transcranial direct current stimulation reduces appetite for risk during ambiguous decision making. J. Neurosci. 27, 6212–6218. 10.1523/JNEUROSCI.0314-07.2007 17553993 PMC6672163

[B16] Fregni F. Liebetanz D. Monte-Silva K. K. Oliveira M. B. Santos A. A. Nitsche M. A.. (2007). Effects of transcranial direct current stimulation coupled with repetitive electrical stimulation on cortical spreading depression. Exp. Neurol. 204, 462–466. 10.1016/j.expneurol.2006.09.019 17113079

[B17] Fregni F. Liguori P. Fecteau S. Nitsche M. A. Pascual-Leone A. Boggio P. S. (2008a). Cortical stimulation of the prefrontal cortex with transcranial direct current stimulation reduces cue-provoked smoking craving: a randomized, sham controlled study. J. Clin. Psychiatry 69, 32–40. 10.4088/JCP.v69n0105 18312035

[B18] Fregni F. Orsati F. Pedrosa W. Fecteau S. Tome F. A. Nitsche M. A.. (2008b). Transcranial direct current stimulation of the prefrontal cortex modulates the desire for specific foods. Appetite 51, 34–41. 10.1016/j.appet.2007.09.016 18243412 PMC3541023

[B19] Gay A. Boutet C. Sigaud T. Kamgoue A. Sevos J. Brunelin J.. (2017). A single session of repetitive transcranial magnetic stimulation of the prefrontal cortex reduces cue-induced craving in patients with gambling disorder. Eur. Psychiatry 41, 68–74. 10.1016/j.eurpsy.2016.11.001 28049084

[B20] Gomis-Vicent E. Thoma V. Turner J. J. D. Hill K. P. Pascual-Leone A. (2019). Review: non-invasive brain stimulation in behavioral addictions: insights from direct comparisons with substance use disorders. Am. J. Add. 28, 431–454. 10.1111/ajad.12945 31513324

[B21] Goudriaan A. E. Yucel M. van Holst R. J. (2014). Getting a grip on problem gambling: what can neuroscience tell us? Front. Behav. Neurosci. 8:141. 10.3389/fnbeh.2014.00141 24904328 PMC4033022

[B22] Hallett M. (2007). Transcranial magnetic stimulation: a primer. Neuron 55, 187–199. 10.1016/j.neuron.2007.06.026 17640522

[B23] Hanlon C. A. Canterberry M. Taylor J. J. DeVries W. Li X. Brown T. R.. (2013). Probing the frontostriatal loops involved in executive and limbic processing via interleaved TMS and functional MRIat two prefrontal locations: a pilot study. PLoS ONE 8:e67917. 10.1371/journal.pone.0067917 23874466 PMC3706588

[B24] He Q. Chen M. Chen C. Xue G. Feng T. Bechara A. (2016). Anodal stimulation of the left DLPFC increases IGT scores and decreases delay discounting rate in healthy males. Front. Psychol. 7:1421. 10.3389/fpsyg.2016.01421 27703440 PMC5028393

[B25] Hodgins D. C. Stea J. N. Grant J. E. (2011). Gambling disorders. Lancet 378, 1874–1884. 10.1016/S0140-6736(10)62185-X 21600645

[B26] Huang Y. Z. Edwards M. J. Rounis E. Bhatia K. P. Rothwell J. C. (2005). Theta burst stimulation of the human motor cortex. Neuron 45, 201–206. 10.1016/j.neuron.2004.12.033 15664172

[B27] Hudgens-Haney M. E. Hamm J. P. Goodie A. S. Krusemark E. A. McDowell J. E. Clementz B. A. (2013). Neural correlates of the impact of control on decision making in pathological gambling. Biol. Psychol. 92, 365–372. 10.1016/j.biopsycho.2012.11.015 23201037

[B28] ISS (2018). Available online at: https://www.iss.it/documents/20126/45616/18_5_web.pdf/1ab76d19-b28e-f3b4-1860-148986a7dce1?t=1581095770863 (accessed May 30, 2020).

[B29] Jansen J. M. Daams J. G. Koeter M. W. J. Veltman D. J. Van Den Brink W. Goudriaan A. E. (2013). E?ects of non-invasive neurostimulation on craving: a meta-analysis. Neurosci. Biobehav. Rev. 37, 2472–2480. 10.1016/j.neubiorev.2013.07.009 23916527

[B30] Klauss J. Anders Q. S. Felippe L. V. Nitsche M. A. Nakamura-Palacios E. M. (2018). Multiple sessions of transcranial direct current stimulation (tDCS) reduced craving and relapses for alcohol use: a randomized placebo-controlled trial in alcohol use disorder. Front. Pharmacol. 9:716. 10.3389/fphar.2018.00716 30018558 PMC6037838

[B31] Koob G. F. Volkow N. D. (2016). Neurobiology of addiction: a neurocircuitry analysis. Lancet Psychiatry 3, 760–773. 10.1016/S2215-0366(16)00104-8 27475769 PMC6135092

[B32] Lantrip C. Gunning F. M. Flashman L. Roth R. M. Holtzheimer P. E. (2017). Effects of transcranial magnetic stimulation on the cognitive control of emotion: potential antidepressant mechanisms. J. ECT 33, 73–80. 10.1097/YCT.0000000000000386 28072659

[B33] Ledgerwood D. M. Petry N. M. (2004). Gambling and suicidality in treatment-seeking pathological gamblers. J. Nerv. Ment. Dis. 192, 711–714. 10.1097/01.nmd.0000142021.71880.ce 15457117

[B34] Lefaucheur J. P. Aleman A. Baeken C. Benninger D. H. Brunelin J. Di Lazzaro V. (2020). Evidence-based guidelines on the therapeutic use of repetitive transcranial magnetic stimulation (rTMS): an update (2014–2018). Clin. Neurophysiol. 131, 474–528. 10.1016/j.clinph.2020.02.003 31901449

[B35] Lefaucheur J. P. André-Obadia N. Antal A. Ayache S. S. Baeken C. Benninger D. H.. (2014). Evidence-based guidelines on the therapeutic use of repetitive transcranial magnetic stimulation (rTMS). Clin. Neurophysiol. 125, 2150–2206. 10.1016/j.clinph.2014.05.021 25034472

[B36] Lefaucheur J. P. Antal A. Ayache S. S. Benninger D. H. Brunelin J. Cogiamanian F.. (2017). Evidence-based guidelines on the therapeutic use of transcranial direct current stimulation (tDCS). Clin. Neurophysiol. 128, 56–92. 10.1016/j.clinph.2016.10.087 27866120

[B37] Liberati A. Altman D. G. Tetzlaff J. Mulrow C. Gøtzsche P. C. Ioannidis J. P.. (2009). The PRISMA statement for reporting systematic reviews and meta-analyses of studies that evaluate health care interventions: explanation and elaboration. PLoS Med. 6:1000100. 10.1371/journal.pmed.1000100 19621070 PMC2707010

[B38] Limbrick-Oldfield E. H. Van Holst R. J. Clark L. (2013). Fronto-striatal dysregulation in drug addiction and pathological gambling: consistent inconsistencies? NeuroImage Clin. 2, 385–393. 10.1016/j.nicl.2013.02.005 24179792 PMC3777686

[B39] Luigjes J. Segrave R. de Joode N. Figee M. Denys D. (2019). Efficacy of invasive and non-invasive brain modulation interventions for addiction. Neuropsychol. Rev. 29, 116–138. 10.1007/s11065-018-9393-5 30536145 PMC6499746

[B40] Luijten M. Schellekens A. F. Kuhn S. Machielse M. W. Sescousse G. (2017). Disruption of reward processing in addiction: an image-based meta-analysis of functional magnetic resonance imaging studies. JAMA Psychiatry 74, 387–398. 10.1001/jamapsychiatry.2016.3084 28146248

[B41] Martini A. Dal Lago D. Edelstyn N. M. J. Grange J. A. Tamburin S. (2018b). Impulse control disorder in Parkinson's disease: a meta-analysis of cognitive, affective, and motivational correlates. Front. Neurol. 9:654. 10.3389/fneur.2018.00654 30233478 PMC6127647

[B42] Martini A. Dal Lago D. Edelstyn N. M. J. Salgarello M. Lugoboni F. Tamburin S. (2018a). Dopaminergic neurotransmission in patients With Parkinson's disease and impulse control disorders: a systematic review and meta-analysis of PET and SPECT studies. Front. Neurol. 9:1018. 10.3389/fneur.2018.01018 30568628 PMC6290338

[B43] Martinotti G. Chillemi E. Lupi M. De Risio L. Pettorruso M. Di Giannantonio M. (2018). Gambling disorder and bilateral transcranial direct current stimulation: a case report. J. Behav. Addict. 7, 834–837. 10.1556/2006.7.2018.85 30264605 PMC6426396

[B44] Martinotti G. Lupi M. Montemitro C. Miuli A. Di Natale C. Spano M. C.. (2019). Transcranial direct current stimulation reduces craving in substance use disorders: a double-blind, placebo-controlled study. J. ECT 35, 207–211. 10.1097/YCT.0000000000000580 30844881

[B45] Moccia L. Pettorruso M. De Crescenzo F. De Risio L. di Nuzzo L. Martinotti G.. (2017). Neural correlates of cognitive control in gambling disorder: a systematic review of fMRI studies. Neurosci. Biobehav. Rev. 78, 104–116. 10.1016/j.neubiorev.2017.04.025 28456569

[B46] Moher D. Shamseer L. Clarke M. Ghersi D. Liberati A. Petticrew M.. (2015). Preferred reporting items for systematic review and meta-analysis protocols (PRISMA-P) 2015 statement. Syst. Rev. 4:1. 10.1186/2046-4053-4-1 25554246 PMC4320440

[B47] Nautiyal K. M. Okuda M. Hen R. Blanco C. (2017). Gambling disorder: an integrative review of animal and human studies. Ann. N. Y. Acad. Sci. 1394, 106–127. 10.1111/nyas.13356 28486792 PMC5466885

[B48] Nitsche M. A. Paulus W. (2000). Excitability changes induced in the human motor cortex by weak transcranial direct current stimulation. J. Physiol. 527, 633–639. 10.1111/j.1469-7793.2000.t01-1-00633.x 10990547 PMC2270099

[B49] Nitsche M. A. Paulus W. (2001). Sustained excitability elevations induced by transcranial DC motor cortex stimulation in humans. Neurology 57, 1899–1901. 10.1212/WNL.57.10.1899 11723286

[B50] Nitsche M. A. Seeber A. Frommann K. Klein C. C. Rochford C. Nitsche M. S.. (2005). Modulating parameters of excitability during and after transcranial direct current stimulation of the human motor cortex. J. Physiol. 568, 291–303. 10.1113/jphysiol.2005.092429 16002441 PMC1474757

[B51] Ouzzani M. Hammady H. Fedorowicz Z. Elmagarmid A. (2016). Rayyan-a web and mobile app for systematic reviews. Syst. Rev. 5:210. 10.1186/s13643-016-0384-4 27919275 PMC5139140

[B52] Pascual-Leone A. Tormos J. M. Keenan J. Tarazona F. Canete C. Catala M. D. (1998). Study and modulation of human cortical excitability with transcranial magnetic stimulation. J. Clin. Neurophysiol. 15, 333–343. 10.1097/00004691-199807000-00005 9736467

[B53] Paulus W. Peterchev A. V. Ridding M. (2013). Transcranial electric and magnetic stimulation: technique and paradigms. Handb. Clin. Neurol. 116, 329–342. 10.1016/B978-0-444-53497-2.00027-9 24112906

[B54] Pettorruso M. Di Giuda D. Martinotti G. Cocciolillo F. De Risio L. Montemitro C.. (2019a). Dopaminergic and clinical correlates of high-frequency repetitive transcranial magnetic stimulation in gambling addiction: a SPECT case study. Addict. Behav. 93, 246–249. 10.1016/j.addbeh.2019.02.013 30798016

[B55] Pettorruso M. Martinotti G. Cocciolillo F. De Risio L. Cinquino A. Di Nicola M.. (2019b). Striatal presynaptic dopaminergic dysfunction in gambling disorder: a (123) I-FP-CIT SPECT study. Addict. Biol. 24, 1077–1086. 10.1111/adb.12677 30226290

[B56] Pettorruso M. Martinotti G. Montemitro C. De Risio L. Spagnolo P. A. Gallimberti L.. (2020). Multiple sessions of high-frequency repetitive transcranial magnetic stimulation as a potential treatment for gambling addiction: a 3-month, feasibility study. Eur. Addict. Res. 26, 52–56. 10.1159/000504169 31665732

[B57] Politi E. Fauci E. Santoro A. Smeraldi E. (2008). Daily sessions of transcranial magnetic stimulation to the left prefrontal cortex gradually reduce cocaine craving. Am. J. Addict. 17, 345–346. 10.1080/10550490802139283 18612892

[B58] Rapinesi C. Del Casale A. Di Pietro S. Ferri V. R. Piacentino D. Sani G.. (2016). Add-on high frequency deep transcranial magnetic stimulation (dTMS) to bilateral prefrontal cortex reduces cocaine craving in patients with cocaine use disorder. Neurosci. Lett. 629, 43–47. 10.1016/j.neulet.2016.06.049 27365134

[B59] Rosenberg O. Klein L. D. Dannon P. N. (2013). Deep transcranial magnetic stimulation for the treatment of pathological gambling. Psychiatry Res. 206, 111–113. 10.1016/j.psychres.2012.09.045 23078873

[B60] Rossi S. Hallett M. Rossini P. M. Pascual-Leone A. Safety of TMS Consensus Group, (2009). Safety, ethical considerations, and application guidelines for the use of transcranial magnetic stimulation in clinical practice and research. Clin. Neurophysiol. 120, 2008–2039. 10.1016/j.clinph.2009.08.016 19833552 PMC3260536

[B61] Rossini P. M. Burke D. Chen R. Cohen L. G. Daskalakis Z. Di Iorio R.. (2015). Non-invasive electrical and magnetic stimulation of the brain, spinal cord, roots and peripheral nerves: basic principles and procedures for routine clinical and research application. An updated report from an I.F.C.N. Committee. Clin. Neurophysiol. 126, 1071–1107. 10.1016/j.clinph.2015.02.001 25797650 PMC6350257

[B62] Sauvaget A. Bulteau S. Guilleux A. Leboucher J. Pichot A. Valrivière P.. (2018). Both active and sham low-frequency rTMS single sessions over the right DLPFC decrease cue-induced cravings among pathological gamblers seeking treatment: a randomized, double-blind, sham-controlled crossover trial. J. Behav. Addict. 7, 126–136. 10.1556/2006.7.2018.14 29463098 PMC6035030

[B63] Sauvaget A. Trojak B. Bulteau S. Jiménez-Murcia S. Fernández-Aranda F. Wolz I.. (2015). Transcranial direct current stimulation (tDCS) in behavioral and food addiction: a systematic review of efficacy, technical, and methodological issues. Front. Neurosci. 9:349. 10.3389/fnins.2015.00349 26500478 PMC4598576

[B64] Schluter R. Daams J. van Holst R. J. Goudriaan A.E. (2018). E?ects of non-invasive neuromodulation on executive and other cognitive functions in addictive disorders: a systematic review. Front. Neurosci. 12:642. 10.3389/fnins.2018.00642 30283294 PMC6156514

[B65] Schluter R. S. Jansen J. M. van Holst R. J. van den Brink W. Goudriaan A. E. (2017). Differential effects of left and right prefrontal high frequency rTMS on resting state fMRI in healthy individuals. Brain Connect. 8, 60–67. 10.1089/brain.2017.0542 29237276

[B66] Shen B. Yin Y. Wang J. Zhou X. McClure S. M. Li J. (2016). High-definition tDCS alters impulsivity in a baseline-dependent manner. Neuroimage 143, 343–352. 10.1016/j.neuroimage.2016.09.006 27608604

[B67] Soff C. Sotnikova A. Christiansen H. Becker K. Siniatchkin M. (2017). Transcranial direct current stimulation improves clinical symptoms in adolescents with attention deficit hyperactivity disorder. J. Neural Transm. 124, 133–144. 10.1007/s00702-016-1646-y 27853926

[B68] Soyata A. Z. Aksu S. Woods A. J. Işçen P. Saçar K. T. Karamürsel S. (2019). Effect of transcranial direct current stimulation on decision making and cognitive flexibility in gambling disorder. Eur. Arch. Psychiatry Clin. Neurosci. 269, 275–284. 10.1007/s00406-018-0948-5 30367243

[B69] Spagnolo P. A. Goldman D. (2017). Neuromodulation interventions for addictive disorders: challenges, promise, and roadmap for future research. Brain 140, 1183–1203. 10.1093/brain/aww284 28082299 PMC6059187

[B70] Steele J. D. Lawrie S. M. (2004). Segregation of cognitive and emotional function in the prefrontal cortex: a stereotactic meta-analysis. Neuroimage 21, 868–875. 10.1016/j.neuroimage.2003.09.066 15006653

[B71] Terraneo A. Leggio L. Saladini M. Ermani M. Bonci A. Gallimberti L. (2016). Transcranial magnetic stimulation of dorsolateral prefrontal cortex reduces cocaine use: a pilot study. Eur. Neuropsychopharmacol. 26, 37–44. 10.1016/j.euroneuro.2015.11.011 26655188 PMC9379076

[B72] Tschernegg M. Crone J. S. Eigenberger T. Schwartenbeck P. Fauth-Bühler M. Lemènager T.. (2013). Abnormalities of functional brain networks in pathological gambling: a graph-theoretical approach. Front. Hum. Neurosci. 7:625. 10.3389/fnhum.2013.00625 24098282 PMC3784685

[B73] van Holst R. J. Sescousse G. Janssen L. K. Janssen M. Berry A. S. Jagust W. J.. (2018). Increased striatal dopamine synthesis capacity in gambling addiction. Biol. Psychiatry 83, 1036–1043. 10.1016/j.biopsych.2017.06.010 28728675 PMC6698370

[B74] Van Holst R. J. van den Brink W. Veltman D. J. Goudriaan A. E. (2010). Brain imaging studies in pathological gambling. Curr. Psychiatry Rep. 12, 418–425. 10.1007/s11920-010-0141-7 20676945 PMC2933850

[B75] Vaughan R. A. Foster J. D. (2013). Mechanisms of dopamine transporter regulation in normal and disease states. Trends Pharmacol. Sci. 34, 489–496. 10.1016/j.tips.2013.07.005 23968642 PMC3831354

[B76] Williams R. J. Volberg R. A. Stevens R. M. G. (2012). The Population Prevalence of Problem Gambling: Methodological Influences, Standardized Rates, Jurisdictional Differences, and Worldwide Trends. Report Prepared for the Ontario Problem Gambling Research Centre and the Ontario Ministry Of Health and Long Term Care. Available online at: http://hdl.handle.net/10133/3068 (accessed May 8, 2012).

[B77] Woods A. J. Antal A. Bikson M. Boggio P. S. Brunoni A. R. Celnik P.. (2016). A technical guide to tDCS, and related non-invasive brain stimulation tools. Clin. Neurophysiol. 127, 1031–1048. 10.1016/j.clinph.2015.11.012 26652115 PMC4747791

[B78] Zack M. Cho S. S. Parlee J. Jacobs M. Li C. Boileau I.. (2016). Effects of high frequency repeated transcranial magnetic stimulation and continuous theta burst stimulation on gambling reinforcement, delay discounting, and stroop interference in men with pathological gambling. Brain Stimul. 9, 867–875. 10.1016/j.brs.2016.06.003 27350401

